# Distribution of melanoma on the body surface.

**DOI:** 10.1038/bjc.1982.51

**Published:** 1982-02

**Authors:** C. D. Holman, B. K. Armstrong


*Br. J. Cancer* (1982) **45**, 317

### *Letters to the Editor*

SIR,—We refer to the article by Dr. I. K. Crombie (1981) on the distribution of malignant melanoma on the body surface. Unfortunately, the author's calculations of the proportional distribution by site of total body melanocytes may be erroneous.

In his Table III, the proportions of body surface area at different sites appear to have been transposed, such that the surface area of the upper limb is given as almost twice that of the lower limb. The reference quoted (Boyd, 1935) was unavailable to us, but the distributions of body surface area given in other studies (Elwood & Lee, 1975; Briele & Das Gupta 1979) suggest that this table should read as follows:

| | Head | Upper limb | Lower limb | Remainder |
|---|---|---|---|---|
| Surface area (% of total) | 7·5 | 19·4 | 38·5 | 34·6 |
| Melanocyte density (cells/mm²) | 1840 | 1160 | 1130 | 890 |
| % of total melanocytes | 13 | 20 | 39 | 28 |

The corrected distribution of melanocytes shown above is quite similar to the site distribution of malignant melanomas in women, particularly as reported by Crombie from the North American Cancer Registries. That a relationship between risk of melanoma and numbers of melanocytes at different sites is more apparent in women than men, may be explained by the comparative lack of body hair in women. The protection from sunlight afforded by varying degrees of hirsutism is, to our knowledge, undocumented. However, if sunlight is a cause of malignant melanoma and body hair is protective, then a relative deficiency of tumours on the lower limb in men in comparison with women, as described by Crombie, would not be unreasonable.

C. D. J. HOLMAN
B. K. ARMSTRONG
*NH & MRC Research Unit in Epidemiology and Preventive Medicine,*
*University Department of Medicine,*
*The Queen Elizabeth II Medical Centre,*
*Nedlands, Western Australia* 6009
9 *October* 1981

REFERENCES

BOYD, E. (1935) *The Growth of the Surface Area of the Human Body.* Minneapolis: University Press.
BRIELE, H. A. & DAS GUPTA, T. K. (1979) Natural history of cutaneous malignant melanoma. *World J. Surg.* **3**, 255.
CROMBIE, I. K. (1981) Distribution of malignant melanoma on the body surface. *Br. J. Cancer,* **43**, 842.
ELWOOD, J. M. & LEE, J. A. M. (1975) Recent data on the epidemiology of malignant melanoma. *Semin. Oncol.*, **2**, 149.


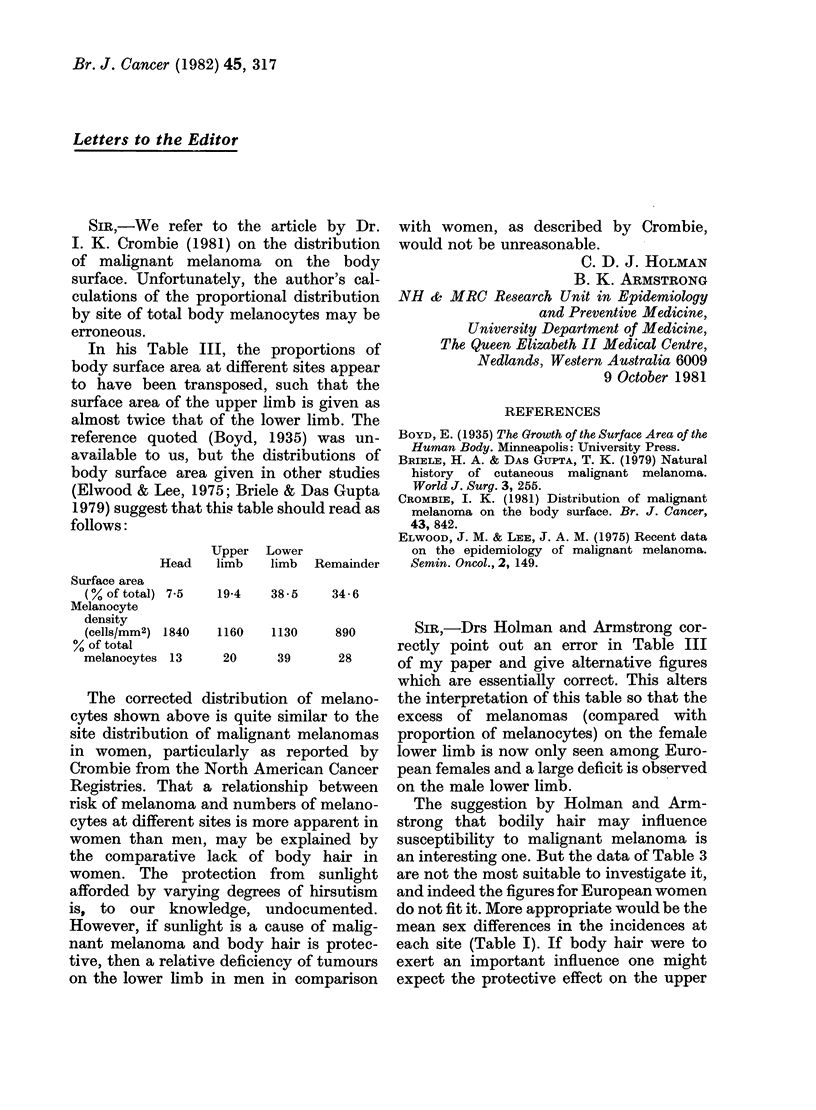


## References

[OCR_00079] Briele H. A., Das Gupta T. K. (1979). Natural history of cutaneous malignant melanoma.. World J Surg.

[OCR_00084] Crombie I. K. (1981). Distribution of malignant melanoma on the body surface.. Br J Cancer.

[OCR_00089] Elwood J. M., Lee J. A. (1975). Recent data on the epidemiology of malignant melanoma.. Semin Oncol.

